# Why Should I Help the Hospitals?

**Published:** 1911-06-18

**Authors:** 


### Why Should I Help the Hospitals?

### SOME REASONS FOR CONTRIBUTING TO-DAY.

The question that heads this article is one that may reasonably be asked by every person on Hospital Sunday. It is well that every one of us should ask himself or herself what are the benefits which the community as a whole, and the individual members of it, are likely to derive from such institutions as our voluntary hospitals, and should ponder, for a moment, the merits of the case for the hospital and the objections raised to our present system. An intelligent interest in the hospitals is the surest means of winning supporters to the fund. Only when you know why you yourself are giving your share towards the fund to-day will you be able to persuade others to follow your example. And it is well to bear in mind that he gives twice who makes his neighbour give.

Suppose for a moment that the existing hospitals are summarily closed. It needs very little stretch of the imagination to picture the chaos and misery that would be caused to thousands of sick people, who are unable to pay for treatment in their own or in private nursing homes, which would inevitably follow such closure. We have grown so accustomed to the hospital that we scarcely think of the general good that it does. We accept it as a matter of course, but we often fail to realise that our voluntary system means the voluntary aid and service and charitable generosity of millions, and that when these millions cease to give such service, generosity, and aid these institutions cannot continue to exist.

The community gains in several ways through the existence of a hospital in its midst. It gains through the treatment of the sick who are cared for and prevented from being a source of danger and difficulty to those that are healthy; through the fact that the institution trains nurses and medical men to acquire the requisite knowledge of disease and the methods of combating it; through the fact that the hospital not only cures disease and alleviates suffering, but prevents both. When you contribute your share towards enabling the hospital to keep up its efficiency and to continue its work, you are therefore doing your part, however small it may be, in helping a national work. At any moment any one of us may need the services of a skilled nurse or medical man; without the experience gained in general and special hospitals a nurse or doctor would be unable to deal efficiently with the grave conditions that are met with every day. The advances in medical and surgical art, which are of incalculable benefit to the community, are the direct results of hospital service. If we had no hospitals, but merely nursing homes, such advances would still be possible, but they would need a much longer time for their development, and they would, it is safe to assert, not be of such general benefit to the community as they are now.

Consider, for instance, the common disease appendicitis. Ten years ago this disease was one of the most dangerous and fatal that afflicted mankind; out of every hundred persons who were seized with it, at least twenty were doomed to death because we did not know the best methods of dealing with it. We do not know now how to ensure recovery in every case, but, mainly through the fact that various methods have been tried and the most careful notes have been taken of the hospital cases, we have attained to such improvements in our methods that the mortality has been greatly reduced. A recent exhaustive analysis of hospital cases has shown that the mortality, even in the grave acute cases, has been reduced to 4 per cent., while hundreds of cases have been treated in succession without a single death. The experience gained in hospital practice has been mainly responsible for this result. And appendicitis is not an isolated instance. Our fever hospitals have taught us much with regard to the proper treatment of acute infectious diseases; the special departments of our general hospitals have enabled us to pay particular attention to certain special diseases, with the result that the general community has benefited to a very large extent.

This is the interested view of the matter, but there remains the more altruistic view. The majority of those who seek hospital aid cannot afford private treatment, especially in surgical cases; by going to a hospital these patients are at least assured of obtaining the best and most modern treatment that we possess. Is it too much to ask that we who are not in want of such treatment, for the time being at least, should give a thought to the sufferings of those who need medical aid and, by contributing our share to the Sunday Fund, enable the hospitals to carry on their work? A thank offering to Providence that we are not as unfortunate as others are, a sympathetic tribute to suffering elsewhere, a fraction that can be spared from our own wealth of this world's goods for the benefit of others who are not so well endowed—this is the altruistic conception of what our share in to-day's contributions really amounts to.

While we are members of a community the health of every unit of that community is a matter of interest to every one of us; it is a foolish fallacy that no one is affected by another's sufferings. The misery produced by disease reacts upon the community both in a hygienic and economic sense. At present it is a counsel of perfection to say that we must strive to abolish disease by preventive measures. We have to take cognisance of the fact that disease is not yet abolished; it is still very much with us, and while we are striving to deal with the causes in other ways, we must not neglect the results. With these results the hospitals are actively concerning themselves, and it is the duty of everyone who is able to do so to help them.

